# Immunosenescence in Choroidal Neovascularization (CNV)—Transcriptional Profiling of Naïve and CNV-Associated Retinal Myeloid Cells during Aging

**DOI:** 10.3390/ijms222413318

**Published:** 2021-12-10

**Authors:** Anja Schlecht, Adrian Thien, Julian Wolf, Gabriele Prinz, Hansjürgen Agostini, Günther Schlunck, Peter Wieghofer, Stefaniya Boneva, Clemens Lange

**Affiliations:** 1 Institute of Anatomy and Cell Biology, Julius-Maximilians-University Wuerzburg, 97070 Wuerzburg, Germany; 2 Eye Center, Medical Center, Faculty of Medicine, University of Freiburg, 79106 Freiburg, Germany; adrian.thien@uniklinik-freiburg.de (A.T.); julian.wolf@uniklinik-freiburg.de (J.W.); gabriele.prinz@uniklinik-freiburg.de (G.P.); hansjuergen.agostini@uniklinik-freiburg.de (H.A.); guenther.schlunck@uniklinik-freiburg.de (G.S.); Stefaniya.boneva@uniklinik-freiburg.de (S.B.); 3 Institute of Anatomy, Leipzig University, 04103 Leipzig, Germany; peter.wieghofer@medizin.uni-leipzig.de; 4 Cellular Neuroanatomy, Institute of Theoretical Medicine, Medical Faculty, University of Augsburg, 86159 Augsburg, Germany

**Keywords:** age-related macular degeneration (AMD), choroidal neovascularization (CNV), aging, immunosenescence, microglia, myeloid cells, RNA-sequencing

## Abstract

Immunosenescence is considered a possible factor in the development of age-related macular degeneration and choroidal neovascularization (CNV). However, age-related changes of myeloid cells (MCs), such as microglia and macrophages, in the healthy retina or during CNV formation are ill-defined. In this study, *Cx3cr1*-positive MCs were isolated by fluorescence-activated cell sorting from six-week (young) and two-year-old (old) *Cx3cr1^GFP^*^/+^ mice, both during physiological aging and laser-induced CNV development. High-throughput RNA-sequencing was performed to define the age-dependent transcriptional differences in MCs during physiological aging and CNV development, complemented by immunohistochemical characterization and the quantification of MCs, as well as CNV size measurements. These analyses revealed that myeloid cells change their transcriptional profile during both aging and CNV development. In the steady state, senescent MCs demonstrated an upregulation of factors contributing to cell proliferation and chemotaxis, such as *Cxcl13 and Cxcl14*, as well as the downregulation of microglial signature genes. During CNV formation, aged myeloid cells revealed a significant upregulation of angiogenic factors such as *Arg1* and *Lrg1* concomitant with significantly enlarged CNV and an increased accumulation of MCs in aged mice in comparison to young mice. Future studies need to clarify whether this observation is an epiphenomenon or a causal relationship to determine the role of immunosenescence in CNV formation.

## 1. Introduction

Age-related macular degeneration (AMD) is the most common cause of blindness in the elderly and can progress in its advanced stages to an atrophic or a neovascular form (nAMD) [1,2,3,4,5]. Though atrophic AMD proceeds slowly, nAMD is often associated with a sudden and rapid loss of central vision. This is caused by the formation of choroidal neovascularization (CNV) growing from the choroid into the subretinal space, which leads to edema, inflammation, fibrosis, and finally irreversible loss of vision (for a detailed review, see [6]). The introduction of intravitreally applicable antibodies targeting vascular endothelial growth factor (VEGF) has led to significant advances in the treatment of nAMD in recent years. However, one-third of patients with nAMD continue to lose vision despite repeated treatments [7,8], highlighting the continuous need to further explore the underlying causes and treatment options of this still-enigmatic disease.

The pathogenesis of AMD and CNV formation is incompletely understood and involves a complex interplay of demographic, genetic, and environmental risk factors [9]. Since aging is the most important risk factor for AMD, cellular senescence—which describes a process in which cells stop dividing and undergo distinct age-related phenotypic changes [10]—is assumed to play an important role in disease development (for a detailed review, see [11]). Therefore, understanding the age-related changes in the retina is of central importance to deciphering its pathogenesis, as well as preventing and treating the disease. Recent evidence has further suggested that the innate immune system is critically involved in the pathogenesis of AMD. As such, variants in genes related to the immune system, such as *CFH, ARMS2/HTRA1* and *C3*, belong to the established risk factors for AMD [9]. Furthermore, myeloid cells, which are part of the innate immune system, accumulate in the subretinal space in the aging eye [12,13,14,15] and have been suggested to contribute to drusen formation in early AMD and CNV in the later stages of disease [16,17,18,19]. Since retinal myeloid cells are long-living and self-maintaining [20,21], they are susceptible to immunosenescence, which refers to the remodeling of and decline in immune efficacy during aging and has been discussed as a contributing factor for AMD [22,23]. However, the age-dependent molecular changes in the myeloid cells of the retina in health and the context of CNV formation are ill-defined.

In this study, we analyzed the transcriptional profile of myeloid cells in the healthy retina and at sites of laser-induced CNV by using young and old myeloid-cell-specific reporter mice and high-throughput RNA-sequencing. Our data revealed that myeloid cells significantly alter their transcriptional profile during physiological aging and that senescent CNV-associated myeloid cells are characterized by the increased expression of cell proliferation and angiogenesis factors, which is associated with increased myeloid cell accumulation and CNV severity.

## 2. Results

**Figure 1:**
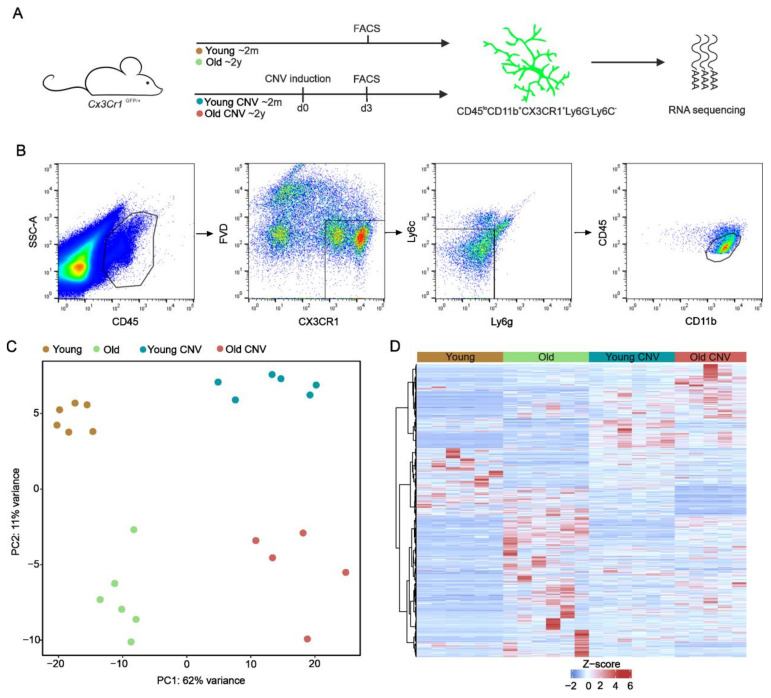
Experimental setup and transcriptional profile of myeloid cells during aging and CNV formation. (**A**) Scheme illustrating experimental setup. Myeloid cells were isolated from six-week-old or two-year-old *Cx3cr1^Gfp/+^* mice, either without laser treatment or three days following laser CNV induction. (**B**) Isolation of myeloid cells via fluorescence-activated cell sorting. Myeloid cells were defined as CD45^low^CD11b^+^*Cx3cr1*-GFP^+^Ly6C^−^Ly6G^−^ and isolated from the retinae of young and old mice without laser induction (young: ocher; old: light green) and three days after laser induction (young: blue; old: red). (**C**) Principal component analysis (PCA) illustrating the clustering of myeloid cells from the four different groups (young control: ocher; old control: light green; young CNV: blue; old CNV: red). (**D**) Heatmap visualizing differentially expressed genes between young and old control groups, as well as between young CNV and old CNV groups. The z-score represents a gene’s expression in relation to its mean expression by standard deviation units (red: upregulation; blue: downregulation).

To decipher the transcriptional profile of myeloid cells in young and aged *Cx3cr1^GFP/+^* mice with and without laser-induced CNV, we isolated myeloid cells, defined as CD45^low^CD11b^+^*Cx3cr1-*GFP^+^Ly6C^−^Ly6G^−^, using fluorescence-activated cell sorting. Following RNA extraction, we performed bulk RNA-sequencing to obtain the quantitative measurements of a total of 29,852 RNA transcripts (Figure 1A,B). Unsupervised analysis displayed a distinct clustering with considerable differences in the expression profiles of myeloid cells between all four groups (young control, *n* = 6 mice; old control, *n* = 6 mice; young CNV, *n* = 6 mice; and old CNV, *n* = 5 mice), with higher discrepancies between the laser and control groups than between the young and old groups (Figure 1C).

### 2.1. Transcriptional Profiling Reveals Changes in Myeloid Cells during Aging

**Figure 2:**
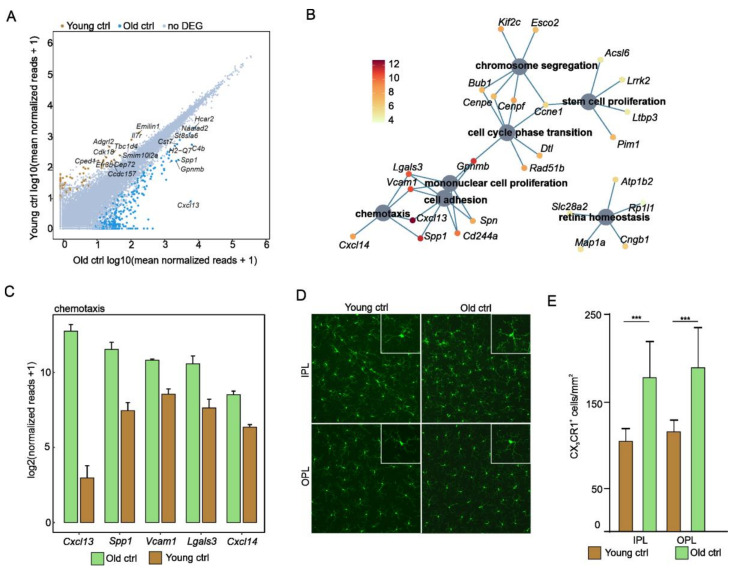
Aging is associated with enhanced proliferation and chemotaxis in retinal myeloid cells. (**A**) Readplot illustrating differentially expressed genes (DEGs) between myeloid cells isolated from young and old mice. The 10 highest expressed genes are labelled (blue dots: highly expressed in old mice; ocher dots: highly expressed in young mice). (**B**) Gene Ontology enrichment analysis of differentially expressed genes. Cnetplot visualizing the top Gene Ontology (GO) biological processes. For simplification, only the top five factors contributing to the respective biological processes are displayed. Color code represents log2 fold change. (**C**) Bar graphs illustrating expression of the top five factors of the GO term chemotaxis. Factors are ordered according to mean expression in old mice. The height of the bar corresponds to the mean of normalized reads, whereas the standard error of mean (SEM) is visualized by the error bars. (**D**) Representative microscopic images of *Cx3cr1*-positive cells from the retinae of old and young mice. A higher magnification of a myeloid cell is shown in each upper right corner. (**E**) Quantification of GFP-positive cells in the retinae of old and young *Cx3cr1*^Gfp/+^ mice. *** *p* ˂ 0.001, *n* = 5 per group. IPL = inner plexiform layer; OPL = outer plexiform layer. Data are presented as mean ± SD. IPL = inner plexiform layer; OPL = outer plexiform layer; DEGs = differentially expressed genes.

Next, we focused on transcriptional changes during physiological aging and compared *Cx3cr1*-positive myeloid cells from the retinae of young and old mice without CNV. Using cutoff criteria (log2FC > 2 or <−2; p. adj. < 0.05), a total of 422 differentially expressed genes (DEGs) were identified, of which 335 were upregulated and 87 were downregulated in myeloid cells of old compared to young mice. The visualization of DEGs is shown as a readplot in Figure 2A. Gene Ontology enrichment analysis demonstrated that DEGs in senescent retinal myeloid cells contribute to processes associated with cell proliferation, such as cell cycle phase transition (GO:0044770; p. adj. = 0.01) and mononuclear cell proliferation (GO:0032944; p. adj. = 0.04), suggesting the enhanced proliferation of aged myeloid cells (Figure 2B). Furthermore, we found a significant enrichment of DEGs in senescent myeloid cells that contributes to migration-associated processes, such as chemotaxis (GO:0006935; p. adj. = 0.004) and retinal homeostasis (GO:0001895; p. adj. = 0.04; Figure 2B). The top five DEGs more enriched in senescent compared to young myeloid cells in the retina were *Cxcl13*, *Spp1*, *Vcam1*, *Lgals3*, and *Cxcl14* (Figure 2C). In particular, *Cxcl13* (young control: 6.8 reads; old control: 6853.1 reads; p. adj. = 9.3 × 10^−32^) and *Cxcl14* (young control: 80.7 reads; old control: 364.6; p. adj. = 5.7 × 10^−14^) were strongly upregulated in myeloid cells isolated from old mice, thus suggesting the activation of migration and recruitment processes in aged myeloid cells. The enrichment of proliferation- and chemotaxis-related genes in senescent myeloid cells was found to be associated with an increase in *Cx3cr1*-GFP^+^-positive cells in the outer and inner plexiform layers of the retina (IPL—young control: 104.0 ± 15.01 cells/mm^2^, old control: 179.8 ± 39.48 cells/mm^2^, *p* < 0.05; OPL—young control: 116.1 ± 12.83 cells/mm^2^, old control: 190.5 ± 45.13 cells/mm^2^, *p* < 0.05; young = 5 mice and old = 10 mice; Figure 2D,E). Finally, we found a greater downregulation of microglial signature genes such as *P2yr12* and *Sall1* in senescent compared to young MCs, whereas others such as *Trem2* and *Tmem119* were similarly expressed in both groups, indicating impaired function in senescent retinal microglia (Supplemental Figure S1).

### 2.2. CNV Induction Changes the Transcriptional Profile of Myeloid Cells in Young Mice

Having established significant age-dependent transcriptional changes in unchallenged retinal myeloid cells, we next focused on changes in the transcriptional profile of myeloid cells challenged by laser-induced CNV. Therefore, we analyzed the transcriptional differences in myeloid cells obtained from young CNV mice compared to the young control group. Using cutoff criteria (log2FC > 2 or <−2 and p. adj. < 0.05), a total of 1135 differentially expressed genes (DEGs) were identified, 771 of which were upregulated following laser CNV induction and 364 of which were downregulated compared with myeloid cells of unlasered young mice (Supplemental Figure S2A). The top differentially expressed genes after CNV induction in the myeloid cells of young mice were leucine TRNA ligase 2 (*Lars2*—young control: 8153.1 reads; young CNV: 37,850.1 reads; p. adj. = 8.8 × 10^−13^), fibronectin 1 (*Fn1*—young control: 23.7 reads; young CNV: 20,506.1 reads; p. adj. = 2.7 × 10^−13^), and secreted phosphoprotein 1 (*Spp1*—young control: 174.3 reads; young CNV: 16,650.9 reads; p. adj. = 1.0 × 10^−21^), also known as osteopontin. Subsequent Gene Ontology enrichment analysis revealed that the DEGs between these two groups contributed to biological processes associated with the proliferation of cells, such as the mitotic cell cycle (GO:1903047; p. adj. = 2.9 × 10^−44^), cell cycle process (GO:0010564; p. adj. = 3.4 × 10^−21^), and chromosome segregation (GO:0098813; p. adj. = 2.8 × 10^−30^) (Supplemental Figure S2B). The top five differentially expressed factors contributing to these GO terms are illustrated in Supplemental Figure S2C–E.

### 2.3. Senescent Myeloid Cells Challenged by CNV Induction Exhibit an Altered Transcriptional Response Associated with Myeloid Cell Accumulation and Increased CNV Size in Aged Mice

**Figure 3:**
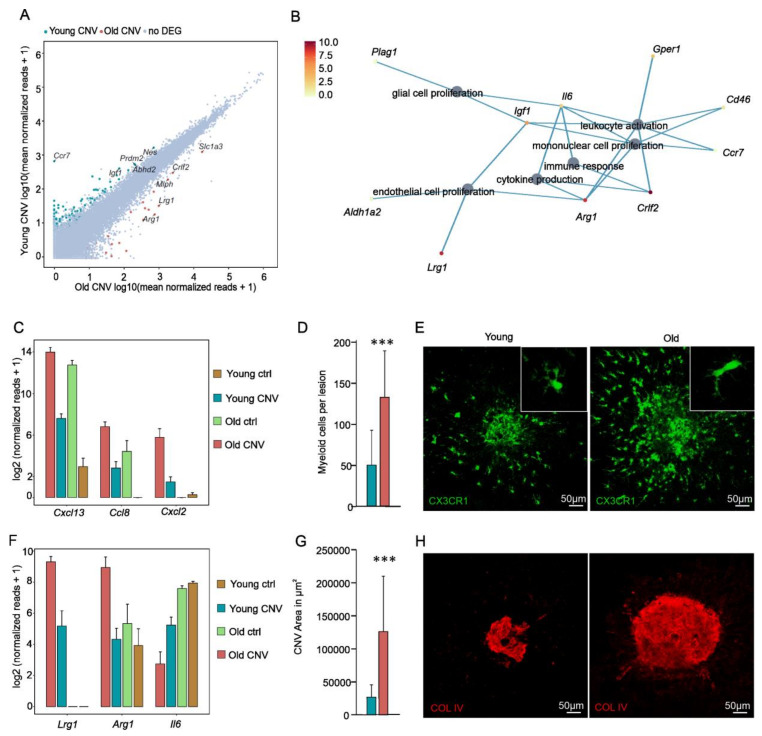
Aging modulates the CNV formation, transcriptional profile, and accumulation of myeloid cells. (**A**) Readplot illustrating differentially expressed genes between myeloid cells isolated from either young or old mice after CNV induction. The top 10 expressed genes are labelled (red dots: upregulated in old mice with CNV; green dots: downregulated in old mice with CNV). (**B**) Gene Ontology (GO) enrichment analysis of differentially expressed genes in aged mice with CNV compared to young mice with CNV without a coincident increase in old control vs. young control. Cnetplot visualizing the top Gene Ontology biological processes. Color code represents mean normalized reads in old mice with CNV. (**C**) Bar graphs illustrating disease-relevant immune-modulatory factors increased in aged mice with CNV compared to young mice with CNV. (**D**) Quantification of Cx3cr1-GFP^+^ myeloid cells accumulating at laser-induced CNV reveals higher cell numbers in aged mice (*n* = 13) compared to young controls (*n* = 13) (d7 after laser induction; *p* = 0.0002). Data are presented as mean ± SD. (**E**) Representative images visualizing an increased accumulation of Cx3cr1-GFP^+^ cells (green) around laser-induced CNV in aged mice. Magnified images of Cx3cr1-GFP^+^ cells are presented in the upper right corner. (**F**) Bar graphs illustrating angiogenesis-related genes with significant changes in the expression level in myeloid cells in aged mice with CNV compared to young mice with CNV. (**G**) The quantification of laser-induced CNV size based on collagen IV staining of RPE/choroidal flatmounts demonstrates significantly enlarged CNV lesion sizes in aged mice (*n* = 15) compared to young controls (*n* = 19) (d7 after laser induction; *p* ˂ 0.001). Data are presented as mean ± SD. (**H**) Representative images of collagen IV stained RPE/choroidal flatmounts (upper panel) and higher magnification of CNV lesions (lower panel) of young (six weeks) and old mice (~two years). *** *p* ˂ 0.001.

Finally, we explored the transcriptional profile of aged myeloid cells in the context of CNV formation. Although we did not observe obvious differences in the morphology of myeloid cells from old or young mice after CNV induction, we found a total of 157 DEGs, 50 of which were upregulated and 107 of which were downregulated in myeloid cells from old mice in comparison to young mice after CNV induction. We found that 31 of these DEGs were also differentially expressed in the myeloid cells of senescent in comparison to young control mice and therefore did not represent CNV-dependent DEGs; rather, they represented age-dependent DEGs. When focusing on the 126 CNV-specific DEGs in senescent myeloid cells, Gene Ontology analysis revealed that they contributed to several immune-associated processes, such as leukocyte activation (GO:0002694; p. adj. = 0.03) and mononuclear cell proliferation (GO:0032944; p. adj. = 0.02) (Figure 3B). The top five factors contributing to enriched GO terms are visualized in Figure 3B. Further analyses demonstrated a significant increase in the expression of several factors relevant to immune cell recruitment and activation in the myeloid cells of aged mice in comparison to young mice following CNV induction. These factors included *Cxcl13* (young CNV: 193.5 reads; old CNV: 16,227.5 reads; p. adj. = 6.5 × 10^−13^), *Ccl8* (young CNV: 6.1 reads; old CNV: 111.6 reads; p. adj. = 0.002), and *Cxcl2* (young CNV: 1.8 reads; old CNV: 54.3 reads; p. adj. = 0.01) (Figure 3C). In line with these gene expression profiles pointing towards enhanced immune cell activation and recruitment, we found considerably more myeloid cells accumulating at CNV lesions in the old group (131.4 cells ± 55.86; *n* = 13 CNV lesions) compared to the young group (49.09 cells ± 41.49; *n* = 13 CNV lesions; *p* ˂ 0.0001) (Figure 3D,E).

Interestingly, CNV-associated senescent myeloid cells expressed more angiogenic factors than young CNV-associated myeloid cells contributing to the GO term endothelial cell proliferation (GO:0001935; *p* = 0.02) (Figure 3B). The most prominent DEGs in this context were leucine-rich alpha-2-glycoprotein 1 (*Lrg1*) and arginase 1 (*Arg1*), which were significantly more upregulated in senescent compared to young myeloid cells associated with CNV (*Lrg1*—old CNV: 630.4 reads, young CNV: 35.1 reads, p. adj. = 1.1 × 10^−4^; *Arg1*—old CNV: 489.7 reads, young CNV: 19.0 reads, p. adj. = 0.01) without showing an increase in expression during physiological aging (Figure 3F). Furthermore, we found a significant downregulation of the angiomodulatory factor interleukin 6 (*Il6)* after CNV induction in old mice (old CNV: 5.69 reads; young CNV: 36.6 reads; p. adj. = 5.73 × 10^−4^). Finally, we determined the CNV size 7 days after laser induction in old and young mice via the quantification of immunohistochemical collagen IV staining on RPE/choroidal flat mounts. In line with the increase of pro-angiogenic factors in myeloid cells of old mice, we found a 5-fold increased CNV lesion size compared to CNV in young mice (old mice: 126.270 µm^2^ ± 83.653, *n* = 15 CNV lesions; young mice: 25.772 µm^2^ ± 19.431, *n* = 19 CNV lesions; *p* ˂ 0.001; Figure 3G,H).

## 3. Discussion

Aging is the main risk factor for age-related macular degeneration and associated CNV formation, which is linked to an activation of the innate immune system [24,25,26]. Immunosenescence has therefore been discussed as a possible mechanism involved in the development of neovascular AMD [22,23]. The present study shows that senescent retinal myeloid cells exhibit a significantly altered transcriptional profile compared to young cells during both during physiological aging and CNV development, which is associated with increased numbers of myeloid cells at sites of CNV and larger CNV lesions in senescent mice.

Recent evidence suggests that retinal microglia comprise a long-living and self-maintaining cell population that is not reconstituted by infiltrating myeloid cells during physiological aging [20,21]. This makes retinal microglia possible candidates for chronic senescence and may suggest that microglial senescence plays a role in age-dependent neurodegenerative diseases of the retina, such as AMD. It has been widely established that resident microglia in the retina and brain undergo various morphological and functional changes during physiological immunosenescence or other cues affecting their homeostasis [27,28,29,30]. Our study shows that retinal microglia cell density increases with age, thus recapitulating the findings of previous studies [13] and revealing that this increase is associated with significant transcriptional changes in senescent microglia. A study by Ma and colleagues who compared the transcriptional profiles of retinal microglia from young and old mice using microarray technology identified 252 genes showing differential expression in 3-month-old MCs in comparison to 12-month-old MCs [31]. Surprisingly, only 2.4% of these 252 genes, such as *Cxcl13*, *Alpl,* and *Chic1,* could be confirmed by our RNA-sequencing analysis to be differentially expressed in senescent microglia. Possible explanations for the different results between our and Ma’s study could include the different ages and strains of the mice studied, the different myeloid cell isolation protocol, and the use of microarray technology by Ma et al., which may be limited by technical issues such as limited probe coverage and inconsistent probe hybridization efficiency. The most significant difference, however, is the different cutoff criteria used in the two studies. While we used a log2FC of >2 or <−2 and an adjusted *p*-value of <0.05, Ma et al. chose to only work with fold-change values and no *p*-value adjustment. Nevertheless, for the sake of completeness, we conducted a reanalysis of the data from Ma et al., comprising the comparison of 3-month-old vs. 24-month-old mice, which were the time points closest to the ones chosen in our analysis. Applying the cutoff criteria used by Ma et al. (uncorrected *p*-value < 0.05 and fold-change > 1.5) for their data and subsequently comparing the resulting DEGs with the DEGs in our analysis resulted in an overlap of 34 genes (Supplemental Table S1). Of note, the reanalysis of the raw data from Ma et al. using the cutoff criteria from our study (log2FC > 2 or <−2, p. adj. < 0.05) resulted in 0 DEGs (data not shown).

On the other hand, our study identified 422 age-dependent genes, such as *Cxcl14*, *Vcam1*, and *Spp1*, that are differentially expressed in senescent retinal MCs and contribute to biological functions such as chemotaxis, cell adhesion, and cell proliferation. In particular, CXCL14 (which synergistically acts together with CXCL13) could contribute to the recruitment of further immune cells in the aged retina [32]. In addition, we found the upregulation of various age-dependent factors, such as *Itgax*, *Lgals3*, *Axl*, *Cst7*, *Clec7a*, and *Spp1* (see Figure 2A–C for *Lgals3* and *Spp1*) in aged myeloid cells, which was in accordance with studies exploring the expression profile of senescent brain microglia [33,34,35,36,37]. 

Our study indicates that in addition to *Lgals3,* further proliferation-associated genes, such as *Vcam1, Spn,* and *Gpnmb*, are strongly enriched in aging retinal microglia cells, which may contribute to the increased myeloid cell density in the retina of aged mice [13]. Although several studies have indicated that aged myeloid cells respond with slowed migration to sites of damage [13], the results of the GO analysis in this study showed that migration-associated genes, such as *Adam8* and *Dpp4,* are enriched in aged myeloid cells. This could reflect the vertical migration of myeloid cells in the retina towards the subretinal space with age, which can be observed in both humans and mice [12,13,14,15]. Finally, *Spp1* emerged as one of the most highly expressed genes in senescent retinal microglial cells and in challenged MCs associated with CNV from young mice. SPP1 binds to multiple integrin receptors that are important for cell adhesion, migration, and survival [38,39] and may thus contribute to the increased cell migration of retinal MCs to the subretinal space during aging and upon laser injury. This is of particular interest because SPP1 has been found to be enriched in the plasma of aged humans and human CNV membranes, as well as to modulate the development of pathological ocular neovascularization in mouse models [19,40,41,42,43]. It is interesting to note that in addition to the GO terms already mentioned, some DEGs of senescent myeloid cells also contribute to retinal homeostasis. In particular, factors expressed on photoreceptors, such as *Cngb1* (cyclic nucleotide gated channel beta 1), *Rp1l1* (retinitis pigmentosa 1 like 1), and *Atp1b2* (ATPase Na^+^/K^+^ transporting subunit beta 2) were observed to be solely upregulated in the myeloid cells of old mice, which may be explained by increased photoreceptor phagocytosis, as previously described [44]. Taken together, the altered transcriptional profiles of senescent retinal microglia in the steady state underline the prevailing notion that retinal microglia proliferate and migrate in aged mice may simultaneously exhibit a compromised ability to interact with and survey their environment, which may translate to an increasing vulnerability to neurodegenerative disease. 

Consistent with this hypothesis, we found a decreased expression of the microglia signature gene *P2ry12* in aged compared to young MCs in the steady state (Supplemental Figure S2) and an increased number of myeloid cells at sites of CNV in old mice, which was associated with larger CNV lesions, as previously described [45,46]. Though Espinosa-Heidmann et al. hypothesized that the presence of age-related dysregulation in choroidal vessel repair after laser injury leads to increased CNV lesions in older mice, Robbie et al. argued that aging results in alterations of the immune status of the choroid and a CCL2-mediated enhanced recruitment of myeloid cells that contribute to the increased formation of CNV [46]. The transcriptional characterization of myeloid cells at sites of CNV in the current study revealed 157 DEGs in myeloid cells from old mice in comparison to young mice after CNV induction. We found that 31 of these DEGs, such as *Prss56*, *Col6a3,* and *Ildr2,* were already found to be enriched in senescent-unchallenged MCs compared to young MCs and could therefore considered to be age-dependent rather than CNV-dependent. The Gene Ontology enrichment analysis of the remaining 126 CNV-associated DEGs in senescent myeloid cells showed that these genes contribute to several immune-associated biological processes, such as leukocyte activation and mononuclear cell proliferation, and may therefore contribute to the observed increased myeloid cell number at sites of CNV in aged mice compared to young mice. The presence of proliferating myeloid cells including microglia was previously shown by us [21]. 

Interestingly, CNV-associated senescent myeloid cells were characterized by an increased expression of pro-angiogenic factors, such as *Lrg1* and *Arg1*, and a decreased expression of anti-angiogenic *IL6*, which have already been related to neovascular diseases in the past [47,48,49,50,51,52]. LRG1, a secreted glycoprotein belonging to the leucine-rich repeat protein family, has been ascribed a distinct pro-angiogenic effect in the literature. Its relevance with respect to neovascular eye disease was first studied by Wang and colleagues, who showed that LRG1 mediates its angiogenic effects in murine models by modulating the TGF-beta signaling pathway in endothelial cells [50]. However, LRG1 has also come into focus in patients with neovascular AMD because elevated levels of LRG1 have been detected in the aqueous humor, vitreous, and CNV membranes of patients with nAMD [53,54,55]. This is of particular interest because the inhibition of LRG1 with a function-blocking antibody successfully inhibits laser-induced CNV in young mice [56], suggesting that anti-LRG1 therapy may be a promising therapeutic option for the treatment of nAMD. Of note, *Vegf* expression in retinal myeloid cells of young and old mice with or without laser-induced CNV was found to be low, which supports previous studies showing that VEGF expression in myeloid cells has no significant effect on CNV formation [17]. Taken together, the transcriptional characterization of senescent myeloid cells following laser-induced CNV suggests the proliferation of myeloid cells and increased expression of angiogenic factors such as *Lrg1* and *Arg1*, which may contribute to increased CNV lesion size in adult mice and indicates that the immunosenescence of myeloid cells may have a direct impact on CNV formation. Further studies on human CNV samples from AMD patients are needed to investigate whether these age-related expression differences in retinal MCs are also found in patients with neovascular AMD and whether the aging MCs cells are a reason for the age-dependency of this disease and contribute to CNV development. These analyses would be crucial for research into efficient strategies and targeted interventions to “rejuvenate” the immune system of AMD patients, and they would thus slow down the progression of the disease and CNV formation.

We acknowledge that this study has several limitations including the use of *Cx3cr1*-GFP reporter mice, which do not allow for reliable differentiation between resident retinal microglia and blood-derived, *Cx3cr1*-positive innate immune cells such as monocytes. However, because microglia are self-maintained, not supplemented by blood-derived monocytes in the steady state, and represent the most significant cell population in terms of cell numbers at sites of laser-induced CNV by far, we consider this limitation to be negligible [19,21,57]. Despite the fact that young mice deficient in *Cx3cr1* were found to have a transcription signature that was comparable to aged mice, we used mice heterozygous for *Cx3cr1* throughout our study to avoid possible effects mediated by *Cx3cr1* expression differences [58]. Nevertheless, future studies are needed to investigate the transcriptional profile of microglia cells at sites of CNV using microglia-specific reporter mice or to employ single-cell RNA-sequencing to determine the expression profile of CNV-associated microglia cells with confidence. 

Ultimately, this study suggests that both physiological aging and CNV formation are accompanied by an upregulation of proliferation and immune-modulatory processes in retinal myeloid cells. Additionally, aged myeloid cells release pro-angiogenic factors following laser-CNV induction, such as *Lrg1*, which might account for increased CNV severity in aged mice. Future studies need to clarify whether this observation is an epiphenomenon or a causal relationship to determine the role of immunosenescence in CNV formation with confidence.

## 4. Material and Methods 

### 4.1. Mice

*Cx3cr1*^GFP/GFP^ mice were crossed with C57BL/6J mice to generate *Cx3cr1*^GFP/+^ mice. Mice were bred on a C57BL/6J background devoid of the *Crb1* mutation. Six-week-old *Cx3cr1*^GFP/+^ mice are referred to as young mice, and two-year and older mice are referred to as old mice. All animal experiments were authorized by the local animal care and use committee under the respective EU, national, federal, and institutional regulations for animal experiments (ethical protocol number G14/89).

### 4.2. Laser-Induced CNV Model

The laser-induced CNV model was used as described previously [19,59,60,61,62]. In brief, mice were anesthetized by the intraperitoneal administration of ketamine hydrochloride (100 mg/kg, Pharmacia & Upjohn, Erlangen, Germany) and xylazine (6 mg/kg, Bayer Vital GmbH, Leverkusen, Germany). Pupillary dilatation was achieved by applying 0.5% tropicamide (Bausch + Lomb, Berlin, Germany) and 5% phenylephrine hydrochloride (URSAPHARM Arzneimittel GmbH, Saarbrücken, Germany). After covering the cornea with a coverslip coated with dexpanthenol eye gel (50 mg/g, Bausch + Lomb, Berlin, Germany), three (for immunohistochemistry) or six laser spots (for RNA-sequencing) were applied to each eye using the VISULAS 532s Laser System (532 nm, 150 mW, 100 µm, and 100 ms, Carl Zeiss, Jena, Germany) in combination with a ZEISS Laser Slit Lamp 532s (Carl Zeiss, Jena, Germany). Only laser spots with the visible formation of vaporization bubbles were included in this study.

### 4.3. Fluorescence Microscopy

After trans-cardial perfusion with phosphate-buffered saline (PBS) and 4% paraformaldehyde (PFA), eyes were fixed in 4% PFA for 1 h on ice and processed for flatmounts, as previously described [50,63]. Anti-mouse collagen IV (Merck Millipore, MAB769, Burlington, NJ, USA) was added overnight in a 1:500 (Collagen IV) dilution at 4 °C. A secondary antibody was applied in a dilution of 1:500 (Alexa Fluor^®^ 647, Thermo Fisher Scientific, Waltham, MA, USA) overnight at 4 °C. Images of whole flatmounts were taken using a Hamamatsu NanoZoomer S60 (Hamamatsu Photonics, Herrsching, Germany). To quantify myeloid cell numbers, confocal images were taken using a Leica SP8 confocal microscope (Leica, Wetzlar, Germany).

### 4.4. Fluorescence-Activated Cell Sorting

Following trans-cardial perfusion with 1× PBS and enucleation, eyes were dissected in ice-cold 1× PBS to isolate the retinae. Only central parts (50%) of the retinae were used for FACS, and the peripheral parts were omitted in order to enrich disease-associated myeloid cells. After tissue homogenization and filtration through a 50 µm cell strainer (Sysmex, Goerlitz, Germany), dead cell exclusion was performed by incubation with fixable viability dye 780 (1:1000, 65-0865-14, eBioscience, Waltham, MA, USA). Anti-CD16/CD32 (Fc) receptor (1:200, 553142, BD Biosciences, Heidelberg, Germany) was used to avoid unspecific binding. Following staining with anti-CD45 (1:200, 103133, BioLegend, San Diego, CA, USA), anti-CD11b (1:200, 17-0112-83, eBioscience, Waltham, MA, USA), anti-Ly6C (1:200, 560593, BD Bioscience, Heidelberg, Germany), and anti-Ly6G (1:200, 560601, BD Biosciences, Heidelberg, Germany) for 20 min at 4 °C, retinal myeloid cells were sorted into an RNA-stabilization reagent (QIAGEN, Hilden, Germany) using a MoFlo Astrios EQ High Speed Cell Sorter (Beckman Coulter, Munich, Germany). Retinal myeloid cells were characterized as CD45^low^CD11b^+^Cx3cr1^+^Ly6C^-^Ly6G-cells. The gating strategy is visualized in Figure 2B (first gate: CD45-positive leukocytes; second gate: exclusion of dead cells and gating for endogenous CX_3_CR1-GFP-positive cells; third gate: exclusion of monocytes (Ly6c) and granulocytes (Ly6g); fourth gate: gating for CD45^low^ myeloid cells). For one final sample, two retinae from one mouse were pooled. 

### 4.5. RNA Extraction 

RNA extraction, RNA library preparation, and RNA-sequencing were performed in collaboration with the Genomics Core Facility “KFB-Center of Excellence for Fluorescent Bioanalytics” (University of Regensburg, Germany), as previously described [64]. In brief, RNA extraction was conducted according to manufacturer’s instructions using the RNeasy Plus Mini Kit (QIAGEN, Hilden, Germany). After pelleting the sample by centrifugation, the RNA-stabilization reagent was removed and replaced by an RLT Plus buffer for lysing retinal microglia. Genomic DNA was selectively and efficiently removed by using gDNA Eliminator spin columns for RNA purification. After adding ethanol to the flow-through, the sample was applied to an RNeasy MinElute spin column to collect RNA. Finally, after washing the column, total purified RNA was eluted in RNase-free water. The quality and integrity of total RNA were assessed with a Agilent 2100 Bioanalyzer in combination with the RNA 6000 Pico LabChip Kit (Agilent, Palo Alto, CA, USA).

### 4.6. RNA-Sequencing

First-strand cDNA was generated using SMARTer Ultra Low Input RNA Kit for Sequencing v4 (Clontech Laboratories, inc., Mountain View, CA, USA). Double-stranded cDNA was amplified with LD PCR and purified with AMPure XP beads. Library preparation was constructed by conforming to the Illumina Nextera XT Sample Preparation Guide (Illumina, San Diego, CA, USA). In brief, 150 pg of input cDNA were tagmented via Nextera XT transposome. The products were purified and amplified with a limited-cycle PCR program to construct sequencing libraries. The libraries were quantified with the KAPA SYBR FAST ABI Prism Library Quantification Kit (Kapa Biosystems, Wobum, MA, USA). Equimolar amounts of each library were pooled for cluster generation on the cBot using the Illumina TruSeq SR Cluster Kit v3. The sequencing run was performed on a HiSeq1000 instrument with TruSeq SBS Kit v3 according to the Illumina HiSeq 1000 System User Guide. Illumina image analysis and base calling were recorded in library base call format (.bcl) before being further converted into Fastq files via the CASAVA1.8.2 software.

### 4.7. Differential Gene Expression Analysis

Sequencing data were uploaded to and analyzed on the Galaxy web platform (usegalaxy.eu), as previously described [43,65]. Quality control was performed with FastQC Galaxy Version 0.72 (http://www.bioinformatics.babraham.ac.uk/projects/fastqc last accessed on 30 July 2020). Reads were mapped to the mouse reference genome (Gencode [66], version M25) with RNA STAR [67] Galaxy Version 2.7.2b (default parameters) using the Gencode main annotation file (Gen-code [66], version M25). Two BAM files for each sample (one for each flow cell) were combined into one BAM file per sample using Merge BAM files Galaxy Version 1.2.0. Reads mapped to the mouse reference genome were counted using featureCounts Galaxy Version 1.6.4 [68] (default parameters) and the aforementioned annotation file. 

The output of featureCounts was imported to RStudio (Version 1.2.1335, R Version 3.5.3). Gene symbols were determined based on ENSEMBL [69] release 100 (mouse genes, downloaded on 1 August 2020). Genes with 0 reads in all samples were removed from the analysis. After principal component analysis [70], differential gene expression was analyzed using the R package DESeq2 Version 1.22.2 (default parameters) [70]. Transcripts with log2fold change (log2FC) > 2 or <−2 and a Benjamini–Hochberg adjusted *p*-value of <0.05 were considered as differentially expressed genes (DEGs). For identification of CNV-specific changes in the transcriptional profile during aging, DEG between young and old control mice were defined at first. In a next step, the same was done for the young CNV and old CNV groups. For bioinformatic analysis, the intersections of these genes were determined and excluded before further analysis. This step was crucial to separate pure aging effects from CNV-associated changes in the expression level of DEGs. Gene enrichment analysis was performed using the R package clusterProfiler 3.10.1 [71]. Heatmaps and Venn diagrams were created using the R package ComplexHeatmap 1.20.0 [72] and VennDiagram 1.6.20 [73], respectively. Other data visualization was performed using the ggplot2 package [74]. Genes involved in angiogenesis and immune response were selected based on the Gene Ontology terms “Angiogenesis” (GO: 0001525) and “Immune response” (GO:0006955) (last accessed on 1 August 2020) [75].

### 4.8. Statistics

For statistical analysis, GraphPad Prism (GraphPad Software, Version 6.0, La Jolla, CA) was used. Data were tested for normality applying the D’Agostino–Pearson Omnibus test. If normality was proven, an unpaired *t* test was applied. If the data were not normally distributed, the Mann–Whitney test was applied. Differences were considered significant at a *p*-value of <0.05.

## Data Availability

Data are available from the corresponding author upon request.

## Supplementary Materials

The following are available online at https://www.mdpi.com/article/10.3390/ijms222413318/s1.
