# Supplementary figures and images for: Immunosenescence in Choroidal Neovascularization (CNV)—Transcriptional Profiling of Naïve and CNV-Associated Retinal Myeloid Cells during Aging
Source: Int J Mol Sci. 2021 Dec 10;22(24):13318. doi: 10.3390/ijms222413318 (PMC8707893; doi:10.3390/ijms222413318)

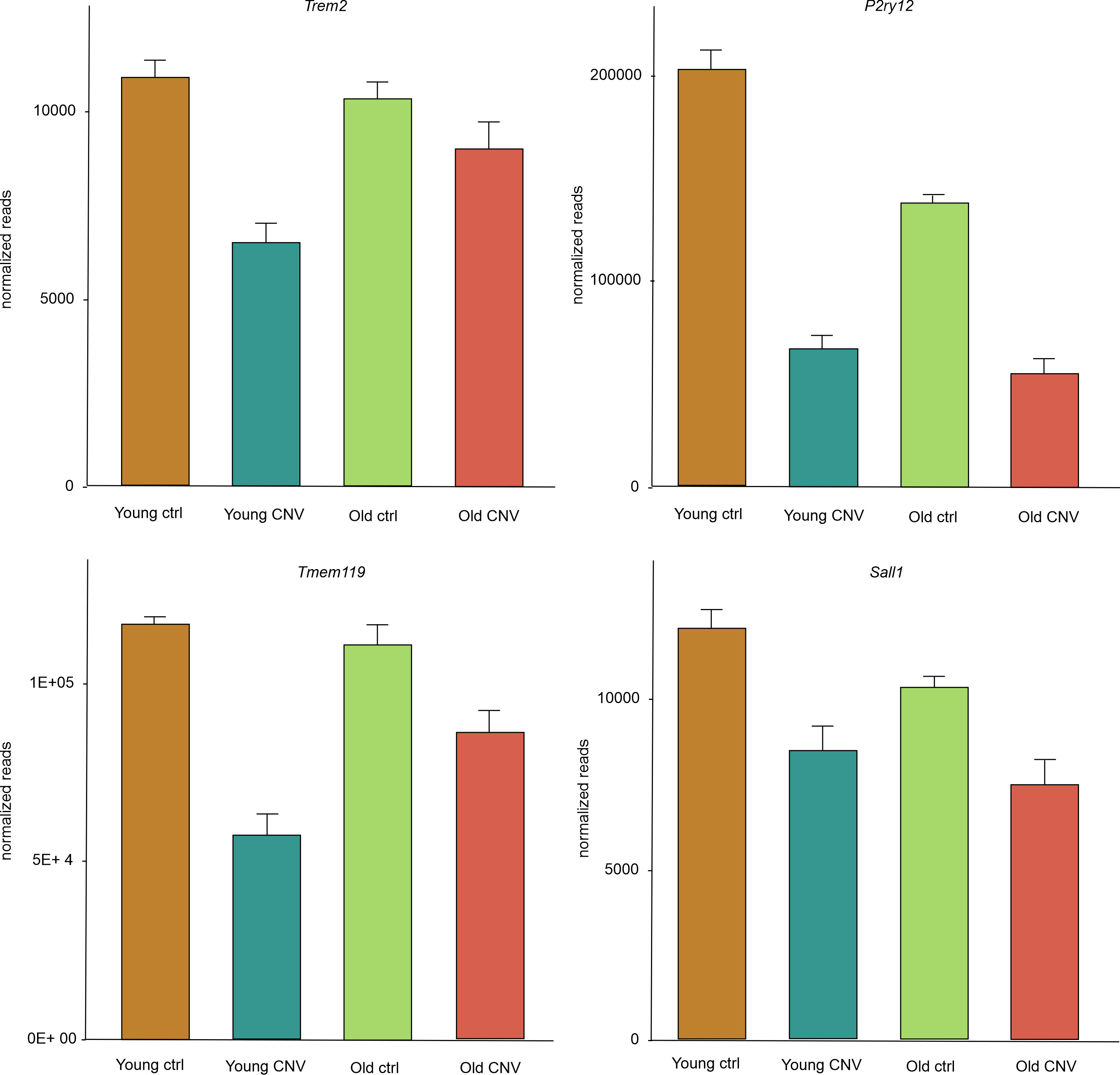

Supplement: Supplementary file 1 [file ijms-22-13318-s001.zip › Supplmental Figure S1.tif]

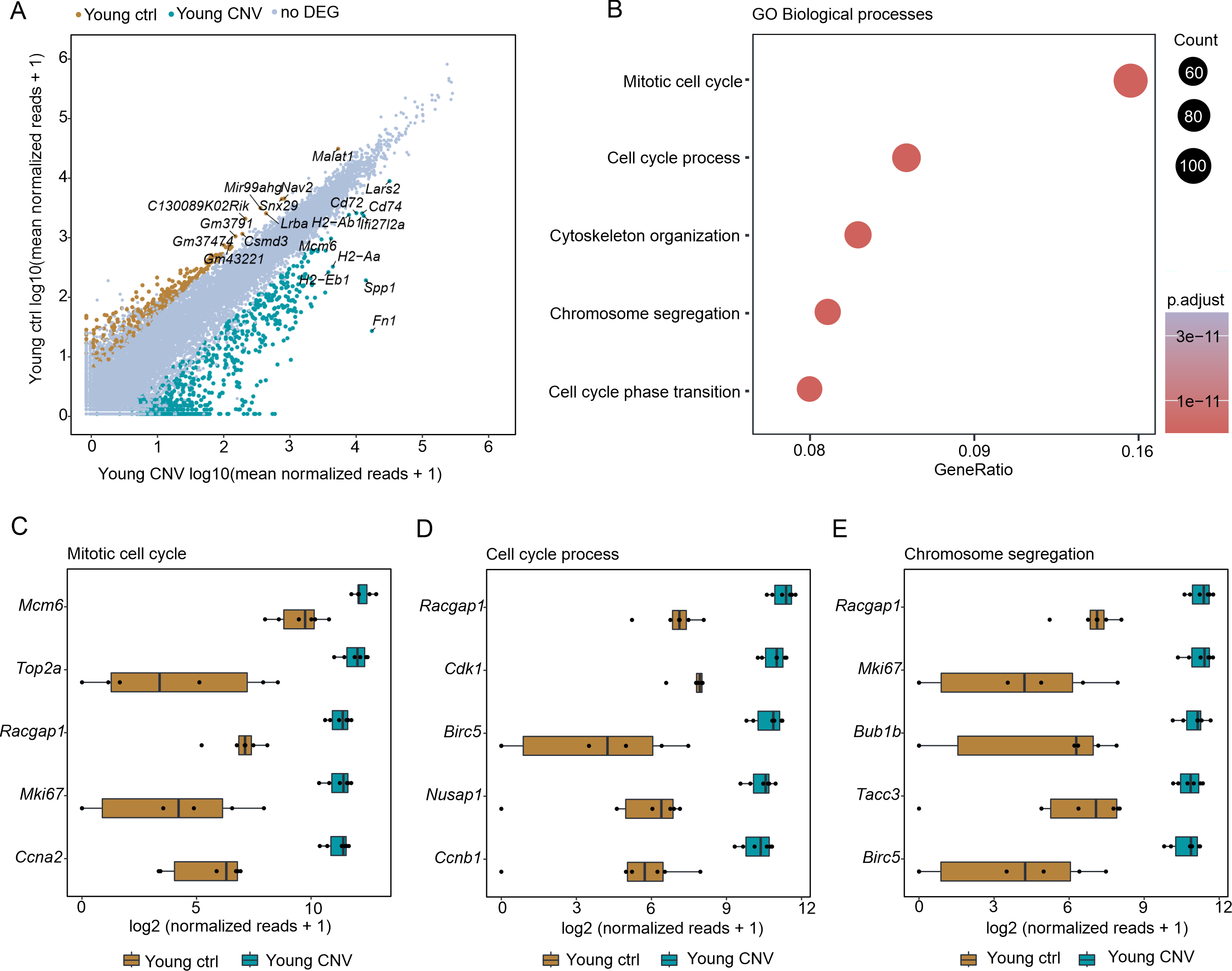

Supplement: Supplementary file 1 [file ijms-22-13318-s001.zip › Supplmental Figure S2.tif]
